# Biomolecular infrared spectroscopy: making time for dynamics

**DOI:** 10.1039/d3sc05223k

**Published:** 2023-11-28

**Authors:** Neil T. Hunt

**Affiliations:** a Department of Chemistry and York Biomedical Research Institute, University of York Heslington York YO10 5DD UK neil.hunt@york.ac.uk

## Abstract

Time resolved infrared spectroscopy of biological molecules has provided a wealth of information relating to structural dynamics, conformational changes, solvation and intermolecular interactions. Challenges still exist however arising from the wide range of timescales over which biological processes occur, stretching from picoseconds to minutes or hours. Experimental methods are often limited by vibrational lifetimes of probe groups, which are typically on the order of picoseconds, while measuring an evolving system continuously over some 18 orders of magnitude in time presents a raft of technological hurdles. In this Perspective, a series of recent advances which allow biological molecules and processes to be studied over an increasing range of timescales, while maintaining ultrafast time resolution, will be reviewed, showing that the potential for real-time observation of biomolecular function draws ever closer, while offering a new set of challenges to be overcome.

## Abstract 2

A perspective discussing the ways in which infrared spectroscopy can be used to study biomolecular processes over a wide range of timescales.

## Graphical abstract

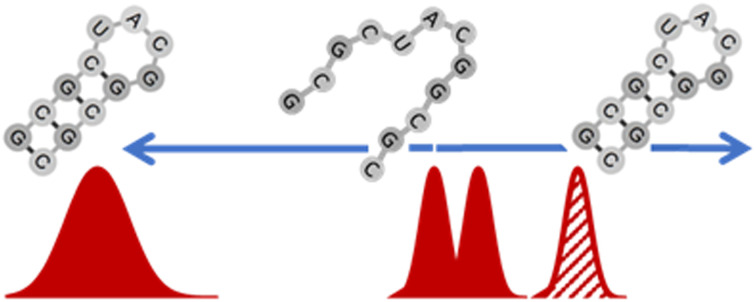

## Introduction

Time resolved infrared (IR) spectroscopy offers many potential advantages for the study of biomolecular systems and processes. Structure is intimately linked to function in biological molecules and IR spectroscopy exploits the absorption of light by vibrational modes involving small groups of atoms so that the frequency, intensity and linewidth of bands in an IR spectrum provide structural information at the level of individual bonds or functional groups. In the case of biomacromolecules such as proteins or nucleic acids, the formation of secondary and tertiary structures, *via* H-bonding for example, creates interactions (couplings) of localised vibrational modes. This coupling leads to new spectroscopic signatures that are diagnostic of the larger 3D motifs, meaning that an infrared spectrum of a biomolecule is effectively a fingerprint of its structure.^1,2^

Under the conditions at which most biological processes occur, ambient temperatures or slightly above in the solution phase, it is important also to consider the impact of dynamic motion on any static picture of the 3D macromolecular structure. Structural dynamics encompass H-bond fluctuations and solvent rearrangements (fs–ps), progressing through amino acid side chain motions (tens of ps) to secondary structure change and diffusive processes (ns–μs). Occurring on increasingly longer timescales are intermolecular interactions, large-scale conformational change, domain-scale reorganisations (μs–ms), aggregation (minutes) and cascade-type processes (hours). Altogether these account for some 15–18 orders of magnitude in time.

While a number of powerful biophysical techniques offer extremely high-resolution structural information, access to dynamics spanning such a wide temporal range represents a major technological challenge. The combination of IR spectroscopy with ultrafast lasers has opened the door to a range of pump-probe type methods that confer the ability to observe biological molecules with time resolution on the order of 100 fs under ambient temperature, solution phase conditions.^3,4^ This means that, in principle, we have the tools to observe the evolution of biomolecular structure on timescales from significantly shorter than the lifetime of a H-bond in solution (∼1 ps) upward to capture the full range of biological processes.

**Fig. 1:**
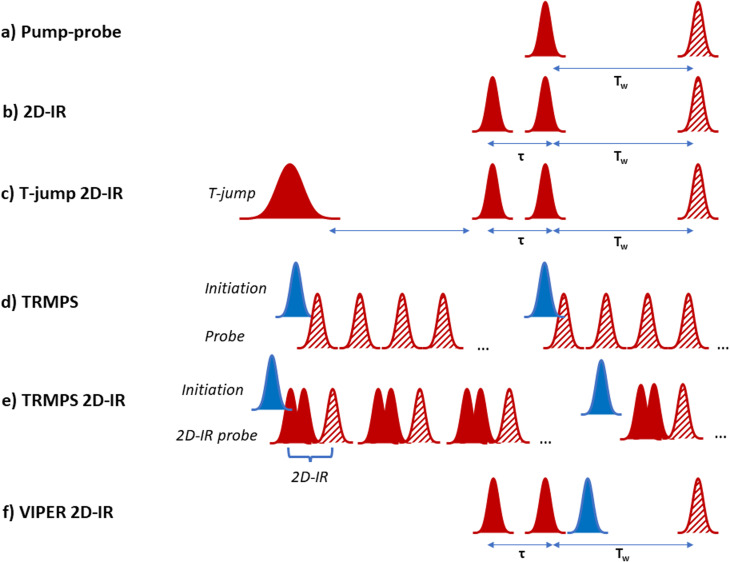
Schematic diagram of laser pulse sequences referred to throughout text. (a) Pump–probe, (b) 2D-IR, (c) T-jump 2D-IR, (d) TRMPS, (e) TRMPS 2D-IR, (f) VIPER 2D-IR. In the diagrams red indicates an IR frequency pulse while blue indicates UV-visible wavelengths. Solid pulses indicate ‘pump’ or ‘initiation’ while hatched pulses indicate ‘probe’ pulses.

Historically, a series of different pump-probe methodologies have evolved which each access specific timescales. Ultrafast IR spectroscopy (Fig. 1a and b) provides high time resolution dynamic information, but the signals arise from the excitation of vibrational modes that have natural lifetimes on the order of a few picoseconds or less. These short lifetimes mean that vibrational energy is rapidly dissipated to a range of low frequency and solvent modes, causing the ultrafast IR signals to decay.^5^ The diminishing signal size places limitations on the processes that IR pump-probe experiments can ‘see’ to those occurring within five times the vibrational lifetime, providing a fundamental barrier to observing slower processes with high time resolution.^6^

At longer timescales, photoinitiation using an ultrashort UV or visible wavelength pulse followed by an IR probe enables experiments up to ns timescales while maintaining high time resolution. These experiments are limited conceptually to light-absorbing systems and practically by the length of the optical delay lines used to control the time between laser pulses.^7^ Nanosecond to microsecond timescales have been accessed by adding a second laser for initiation with electronic control of the timing of the second probe laser.^8^ This approach has enabled both light and temperature jump activation and access to longer times, but at the expense of picosecond dynamics. The millisecond range and upward is well-served by a broad range of stopped flow, continuous flow or step-scan/rapid scan type IR spectroscopies, but with very limited time resolution.^9,10^ Each of these methods adds valuable insight but, as the equipment is costly, very few laboratories have access to all experiments meaning that measuring the same process on the same sample with multiple different methods has been a logistical challenge, while stitching together dynamics obtained in different ways reliably is a difficult undertaking.

Despite these challenges, it has been clear that major benefits could accrue from developing the ability to cover timescales seamlessly. Principal among these is the access to information relating to connectivity between processes that occur on disparate timescales. An excellent example is provided by the problem of allosteric communication in proteins, or nucleic acids, whereby an event in one part of a biomolecule structure, such as ligand binding, influences behaviour in another, remote part of the structure, for example by modulating binding affinity. The mechanisms by which this allosteric communication proceed remain unclear, but one possibility lies in the transmission of dynamic effects through the structure.^11^ Unravelling such communications requires the ability to extend ultrafast time resolution to a large range of other timescales which monitor the different stages of the allosteric process.

In this Perspective, the intention is to review recent developments in time-resolved IR spectroscopy methods that allow measurement of biomolecular systems seamlessly across a wide range of timescales. The focus will be on the current state of the art in terms of laser technology and the use of multidimensional spectroscopies, showing how their useful time range has been expanded by a range of methods. The review will also include exciting progress towards augmenting spectroscopic methods *via* the use of molecular tools and local structural probes that provide close control and detailed localised insight. To maintain this focus, the interested reader is directed to many comprehensive foregoing review articles relating to the development and use of ultrafast IR spectroscopies for studying molecular dynamics that provided the firm foundations for the work described here.^12–27^ Finally, an outlook will be presented along with consideration of the potential of these methods for providing insight into functional biological systems in the future.

## Extending the reach of IR methods

An important consideration when following biomolecular processes using time resolved spectroscopy is identifying a means of beginning the process or ‘starting the clock’. Here we focus on three different approaches, the use of a rapid change in temperature (T-jump) or a pulse of UV or visible wavelength light to achieve initiation as well as a method that uses rapid scan data acquisition to follow processes occurring over minutes or hours.

### T-jump

The use of T-jump initiation has many benefits for the study of biological systems, the chief among which is that the perturbation used to initiate the process being studied exploits the natural potential energy surfaces of the molecule, and so it can, in principle, be applied to any system of interest. The method employs a laser pulse, tuned to an absorption band of the solvent, to deliver sufficient energy to cause a local jump in temperature, typically on the order of 10 K. The resulting evolution of the system in response to the T-jump can then be followed using a variety of probe methods, from simple absorption changes to more complex multidimensional spectroscopies (Fig. 1c).^19,28–30^

Practically, T-jump studies exploit nanosecond pulsed lasers to deliver the initiation pulse *via* excitation of an overtone band of D_2_O.^29^ The latter is often used as a solvent for biomolecule IR studies to avoid the overlap between the H–O–H bending vibrational mode of H_2_O occurring at 1650 cm^−1^ and the key absorption bands that are diagnostic of biomolecule structure, such as the amide I band of proteins (the C=O stretching mode of the peptide link) and base vibrational modes of nucleic acids.^1,2^

**Fig. 2:**
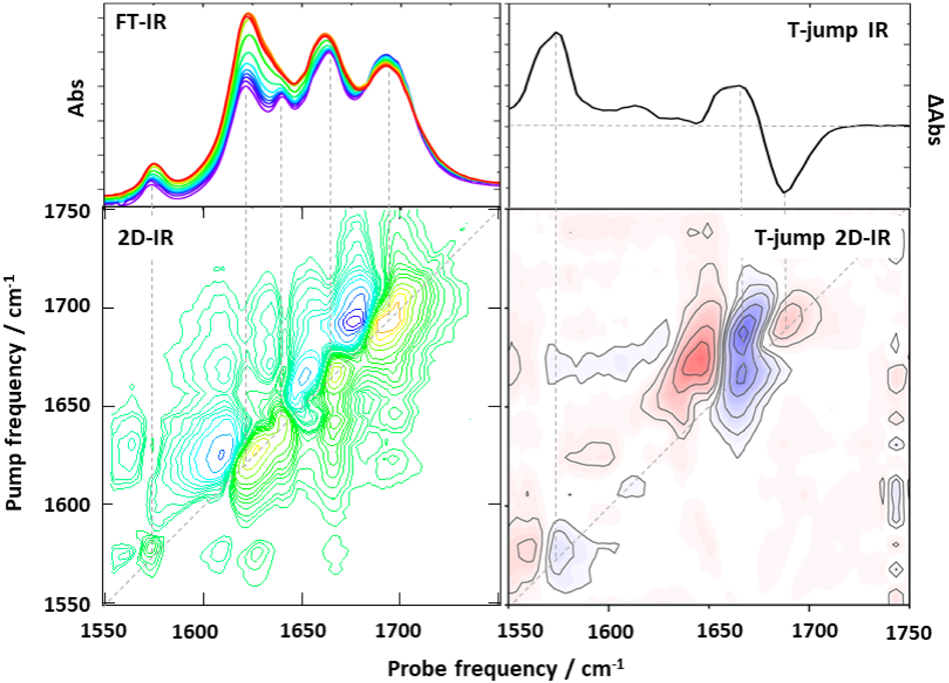
Exemplar comparison of data obtained using one and two dimensional spectroscopy experiments. (Left hand column) Comparison of IR absorption spectroscopy (top) with 2D-IR (bottom) of a double-stranded DNA oligomer obtained under equilibrium conditions. The additional information obtained from the use of 2D-IR *via* off-diagonal peaks highlighting the intra and intermolecular coupling of base vibrational modes of the DNA due to intrinsic base structure and Watson Crick H-bonding and stacking interactions is apparent in the lower spectrum. (Right hand column) Comparison of non-equilibrium T-jump IR spectrum showing melting of a double stranded DNA oligomer (top) with a T-jump 2D-IR spectrum (bottom), both obtained 30 μs after melting was initiated with a T-jump pulse. In both columns dashed lines show the relationships between features in the 1D-spectrum and features appearing on the 2D-IR spectrum diagonal. Additional off-diagonal peaks in the T-jump 2D-IR spectrum provide information on changes in couplings and inter-relationships of spectral features which overlap in 1D.

The first combination of T-jump initiation with non-linear IR spectroscopy was reported in 2005, with 2D-IR spectroscopy used to follow the unfolding of ubiquitin over timescales from nanoseconds to milliseconds (Fig. 1c).^31^ The use of 2D-IR or dispersed vibrational echo spectroscopy to probe the dynamics of the protein allowed a more detailed insight into the evolution of the amide I band than would have been possible using a straightforward IR absorption probe *via* the access that multidimensional spectroscopies give to the coupling patterns that underpin the biomolecule line shape (exemplified in Fig. 2). T-jump 2D-IR methods have since been applied to observe temperature-induced dynamics in a range of systems from secondary structure changes in peptides,^32,33^ and proteins^34–36^ to DNA hybridisation^37–42^ and tautomerisation kinetics.^43^ Most recently, studies have been made of DNA-based systems including abasic sites to mimic lesions.^37^ These studies identified the destabilising impact of missing bases in a double stranded sequence and in particular showed the ability of IR spectroscopy to provide location specific information on behaviour on either side of the lesion *via* the characteristic absorption band profiles of the individual bases.^44^

Evolution of the T-jump method has been defined by advancements in laser technology. The fundamental need to deliver a relatively large amount of energy to the solvent in a short space of time to produce the initial T-jump has limited the initiation method to nanosecond-pulse-duration lasers, which in turn restricts the time resolution. However, in practice this is not a major restriction as the molecular dynamics of interest that follow a T-jump, such as conformational change, generally occur on ns-timescales and longer, with effects on shorter timescales being limited to solvent or H-bond-related processes. At the opposite end of the temporal spectrum, the maximum time that can be probed after the T-jump is determined chiefly by the cooling dynamics of the sample. The pulse repetition rate of the T-jump laser also limits the maximum accessible time range *via* the arrival of the next initiation pulse, but it is relatively easy to reduce the repetition rate of initiation, by optical chopping of the T-jump laser for example. One of the major challenges for T-jump methods arises from the relatively long data acquisition times when using, for example, an initiation pulse with tens of Hz repetition rate and a probe laser system operating at 1 kHz.^45^ Under these conditions long-time drift of laser outputs can reduce signal to noise ratios, especially at longer delay times where signals are smaller.

A variation of the T-jump strategy employed a higher repetition rate T-jump initiation laser based on a ns-Nd:YAG-pumped optical parametric oscillator operating at 1 kHz alongside a probe laser system based on 10 kHz repetition rate Ti:sapphire technology.^46^ This approach was applied to study the impact of ligand binding on double stranded DNA, showing that the binding of a ligand to the minor groove of the DNA not only slowed down the melting process of the double helix in line with ligand binding affinity, but also suppressed end fraying of the strand outside of the main binding site.^47,48^ In the case of the latter observation, base-specific signals were used to identify an allosteric impact on the dynamics of bases remote from the binding site.^47^ The use of higher repetition rate lasers in this experiment significantly reduced data acquisition times. However, the lower intensity of the T-jump pulses in comparison to systems operating in the tens of Hz range required the initiation process to exploit the OD-stretching vibration of the solvent rather than the more normally used overtone bands. The use of more intense solvent bands to achieve the T-jump necessitates a shorter path length sample and, as cooling proceeds mainly through the CaF_2_ windows of the sample cell, the cooling of the initial T-jump occurs on hundreds of microsecond timescales rather than milliseconds, reducing the maximum observable timescales.^46^

**Fig. 3:**
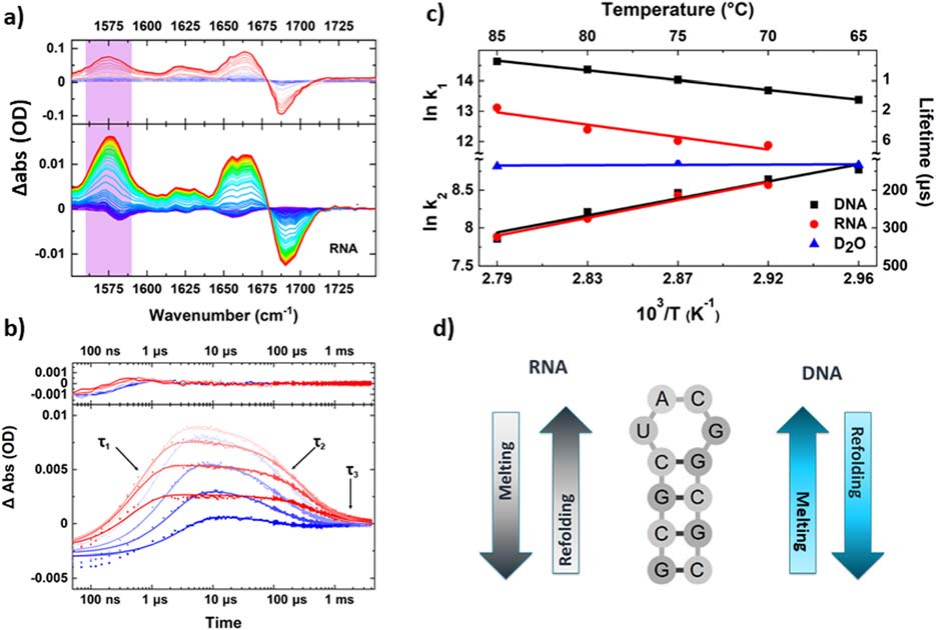
Exemplar data obtained from a transient IR spectroscopy experiment obtained over many orders of magnitude in time. (a) T-jump IR data obtained for unfolding of an RNA tetraloop hairpin. (b) Temporal dynamics extracted from T-jump IR spectra by following the amplitude changes of a particular peak in the spectrum. Note the logarithmic time axis. Three clear timescales are visible in the data including the rising signal assignable to loop melting, and two cooling timescales assigned to refolding of the tetraloop (see text). (c) Shows an Arrhenius analysis of the melting and refolding dynamics, note the apparent negative activation energies obtained for refolding resulting from the complex potential energy landscape of the folding nucleation step. (d) Summary of the mechanisms of melting and refolding obtained from location of site-specific probe groups into the stem of the loops in RNA and DNA, which showed opposing mechanisms for the two molecules.^51^

Using this higher repetition rate T-jump methodology thus brings advantages in terms of signal to noise ratio, but at the expense of access to slower processes such as protein folding, which become restricted to the earliest steps in the process, as was shown by observation of helix destabilisation in calmodulin.^49^ The rapid sample cooling rate does however confer a significant additional benefit because it has been shown to allow direct experimental access to refolding dynamics.^50^ This was demonstrated using RNA and DNA tetraloop structures where both the melting and refolding timescales were found to show Arrhenius temperature-dependencies (Fig. 3).^50^ Refolding dynamics have hitherto been inferred from T-jump melting studies because sample cooling times that extend to millisecond timescales mean that the solvent cools more slowly than the perturbed molecules refold. In contrast, the faster cooling time of the short path length T-jump approach meant that the refolding molecules were not able to equilibrate on the same timescale as the rapidly cooling solvent, leading to the equivalent of a combined temperature jump – temperature drop experiment. Examination of the cooling dynamics of the tetraloops showed that while the refolding times of RNA and DNA hairpins were similar, the melting time of RNA was significantly longer, which was attributed to stronger base stacking interactions in the base paired RNA stem.^50^ A follow-up study using different base sequences in the stem of the loop to identify site specific melting and refolding dynamics produced evidence for RNA and DNA loops melting and folding *via* different mechanisms, with end fraying in DNA leading to stem unzipping in contrast to RNA sequences which showed no end fraying and appeared to melt from the loop downwards.^51^

Direct access to refolding times is beneficial because it is often the folding or binding processes that are biologically relevant, whereas T-jump initiation methods tend to drive the reverse process (unfolding, dissociation). However, there is also a clear need to extend T-jump spectroscopy to much longer timescales to allow access to biologically important processes such as domain-scale restructuring, folding of large proteins or downstream changes which result from the one first initiated. For example, one could envisage observing structure changes following T-jump initiated ligand dissociation or situations where T-jump initiated structure change leads to ligand binding. Such examples would open up the possibility of differentiating induced fit from conformational selection type intermolecular interactions or tracking allosterically driven events.

A solution to this problem was demonstrated in 2020 with the use of intensity-modulated optical heating.^52^ Here, the T-jump was provided by a continuous wave (CW) fibre laser and acousto-optic modulator (AOM) combination. The latter was used to control the light incident on the sample which provided the heating. By combining a rapid initial spike (rise time 300 ns) with a lower level, continuous heating it was possible to induce a rapid rise in temperature in the sample, with a flat-topped profile which could then be sustained, in principle indefinitely, by balancing heat input from the laser with energy loss from the sample. The method was demonstrated for use with proteins, using ubiquitin, and DNA, *via* a first study of i-motif DNA unfolding.^52^ For ubiquitin, the longer timescale experiments provided access to unfolding of β-sheet structures on 10–100 ms timescales, beyond the reach of earlier methods. Access to such long timescale processes also meant that the dynamics associated with refolding were now found to lag behind the solvent cooling, but it was reported that the convolution of timescales made the absolute dynamics hard to extract. When applied to i-motif DNA, 2D-IR probing allowed observation of processes related to unfolding on 100 ms timescales.^52^

### Photoactivation

A commonly-used alternative to T-jump initiation is photoactivation. In this a UV or visible laser pulse is used either to initiate a natural electronic transition of the biomolecule, for example in proteins which contain chromophores, or to excite an artificially implanted light activated moiety to drive a structural or energetic process.^7,8,53–56^ One advantage of light activation occurs from the higher time resolution measurements that are possible. In contrast to T-jump, excitation of the target molecule is achieved directly and *via* more strongly absorbing electronic transitions and so can be achieved with ultrafast laser pulses, as opposed to the need for higher energy, nanosecond pulses in T-jump experiments. The excitation of a specific chromophore also means that the initiation process is spatially-localised and this has been exploited for a range of experiments exploring allosteric signal transduction and energy transfer pathways.^57^

#### Methodology development

The first example of transient 2D-IR spectroscopy was reported in 2003 when a 420 nm pulse was used to excite a transition of an azobenzene-based photoswitch attached to a short chain cyclic peptide.^58,59^ The photoswitch triggered a conformational change, the results of which were then followed using 2D-IR experiments as a time delayed probe. Time delays of up to 1.7 ns following excitation were studied, while the excitation pulse duration was 700 fs, leading to time resolution on the picosecond timescale.^59^ Subsequent development of this method *via* the use of synchronised lasers gave access to picosecond to microsecond temporal ranges by using electronically controlled delay times between pulses from the two lasers.^60^ Thereafter the method of time resolved multiple probe spectroscopy (TRMPS) was reported, which employed two lasers with different pulse repetition rates (Fig. 1d and e).^61^ In TRMPS experiments, the photoactivation is provided by the lower repetition rate laser with the higher rate laser being used to provide multiple probe events before the next activation pulse arrives. This method was combined with optical delays to provide continuous scanning from picoseconds to milliseconds and has since been extended to high repetition rate Yb-based lasers providing femtosecond to second spectroscopy.^62^ More recently, methods which can, in principle be used to synchronise any laser systems have been reported, with a view to allowing laboratories with existing equipment to extend measurements to longer timescales,^63^ while the use of high repetition rate lasers was also used to enable microsecond to millisecond transient-2D-IR spectroscopy.^64^ Overall, this array of technological approaches to studying light activated processes in biomolecules has provided a range of solutions that can deliver femtosecond to picosecond time resolution extending over seconds of observation time. Together they bring about the exciting possibility of seamless reaction following. In the next section, applications of this technology to biomolecular systems will be reviewed.

#### Insights into allostery

The underlying mechanisms of allosteric communication in proteins and nucleic acids has been an ongoing topic of debate, with increasing evidence for a role for structural dynamics in signal communication.^11^ Time resolved IR spectroscopy over a broad temporal range is well placed to contribute to this problem, given that vibrational dynamics occur on picoseconds while the allosteric effect may involve structure change or ligand binding/release occurring over many milliseconds.

An investigation into the transmission of vibrational energy through protein structures was carried out using site specific labels, both of which were modified alanine amino acids.^57^ One was derivatised with an azulene based chromophore, to act as a donor of vibrational energy, while the second was modified to contain an azide unit which was used as a reporter group. The essence of the experiments was to inject vibrational energy into a specific point on a β-hairpin peptide and use the time of arrival of the energy at the acceptor group to measure rates of energy transmission, achieved by monitoring the IR signature of the azido group. By changing the relative positions of the donor and acceptor it was determined that the vibrational energy transfer rate along the peptide backbone was faster than through H-bond contacts, however in systems such as β sheets, the transfer of energy between strands outperformed that *via* a route that follows the backbone, because the latter, although by a faster mechanism, involves a longer route.^57^

A sequence of studies has been carried out using time resolved IR spectroscopy on PDZ domains, which play roles in protein interactions. It was reported that an azobenzene-derived photoswitch could be covalently linked in such a way as to bridge a binding groove of the PDZ domain and so be used to imitate a structural change that occurs naturally to the domain upon ligand binding.^65,66^ Time resolved IR studies showed that the action of the photoswitch induced opening of the binding groove on 100 ns timescales, much slower than the rapid change in structure of the photoswitch and indicative of the dynamic motion of the groove structure in response to the perturbation.^65^ The time dependence of the dynamics was found to be nonexponential. By combining the spectroscopic results with molecular dynamics (MD) simulations it was concluded that this is due to the motion of water molecules in response to the change in structure of the protein. This led to the suggestion of a role for water molecules in both the protein dynamics and allosteric effects of the PDZ domain.^65^

A later study of a PDZ domain extended the results of this study in both scope and timescale. In this study, the azobenzene photoswitch was instead placed on a peptide ligand of the domain, such that a light activated change in photoswitch conformation led to a change in peptide structure.^67^ By optimising the position of the photoswitch it was possible to alter the binding affinity of the peptide for the domain by a factor of five upon excitation, such that binding could be light controlled with loss of binding leading to a subsequent change in the structure of the domain and an allosteric change in binding affinity at a distant site. This approach had the significant benefit of allowing the peptide ligand to carry the photoswitch, leaving the PDZ domain in an unmodified and so more natural form. In practice, the concentrations of the protein and peptide required for the experiments in combination with the binding affinities in the two states meant that, while the peptide structure was changed by the photoswitch, the peptide did not change its bound state. Despite this, the perturbation in structure was sufficient to observe the related change in the structure of the domain, providing valuable new insights. Furthermore, by selectively isotope labelling the peptide and protein it was possible to cleanly separate the contributions to the signal from each. Once again, MD simulations were used to enhance the interpretation of the data allowing the free energy landscape of the PDZ domain to be described as a series of metastable states, albeit separated by relatively small changes in structure.^67^ The latter observation was postulated to result in part from the 42 μs time limit of the apparatus used, with subsequent steps expected to occur on ms timescales.

In a further study of the PDZ system, the azobenzene photoswitch was positioned on an allosterically active α-helix of the domain, such that activation initiated a structural transition and ultimately to a change in binding affinity for a peptide in a remote binding pocket. It was thus observed that the photoswitch-induced changes propagated through the PDZ domain leading to changes at the peptide position in around 200 ns, leading to a quantifiable estimate of the speed of allosteric signal transduction.^68^

The versatile azobenzene photoswitch that provided such a broad range of different perspectives on PDZ domain behaviour *via* changes in its position within the allosteric protein system has also been used to study the MCL-1 (myeloid cell leukaemia) protein and its complexes.^69,70^ The MCL-1 protein is part of a wider family of proteins (BCL-2) which control cell apoptosis *via* a complex suite of protein–protein interactions. Part of the MCL-1 mechanism involves the binding of α-helical peptide within a groove in the protein structure. The machinery of these interactions is not well understood, for example the peptide ligand is known to be disordered in solution and the precise role of induced fit or conformational selection processes, which determine whether the peptide forms its bound structure before or after interaction with the protein, are yet to be determined. Additionally, the way that binding-induced structural rearrangements arise from, or influence, allosteric effects are still unknown. In a pair of studies, an azobenzene photoswitch was placed on a number of helical peptides bound to the MCL-1 protein.^69,70^ Activation of the photoswitch led to a perturbation of the helical structure. While this did not induce dissociation, time resolved IR spectroscopy was able to follow structural rearrangements of the protein in response to the switched peptide structure. From this it was concluded, similarly to the PDZ domain behaviour, that a cascade of structural rearrangements of the protein follow the perturbation of the peptide, occurring on times from pico- to microseconds. It was further determined that the peptide binding follows an induced fit mechanism, where initial contacts are followed by a rearrangement of the binding partners into the bound state. It was proposed that the protein rearrangements may form the basis of an allosteric signal arising from the binding.^69^ The subsequent study investigated a wider range of peptides finding a substantial variation in the timescales observed for the structural rearrangements following photoswitch activation.^70^ These were linked to differences in specific peptide–protein interactions occurring upon the rearrangement pathway following the perturbation. As the MCL-1 protein can bind to a diverse range of partners such dynamic differences may shed light on how peptide structure and binding interrelate offering the potential for informing anticancer strategies for small-molecule manipulation of the apoptosis pathway.

#### Intermolecular interactions and binding

**Fig. 4:**
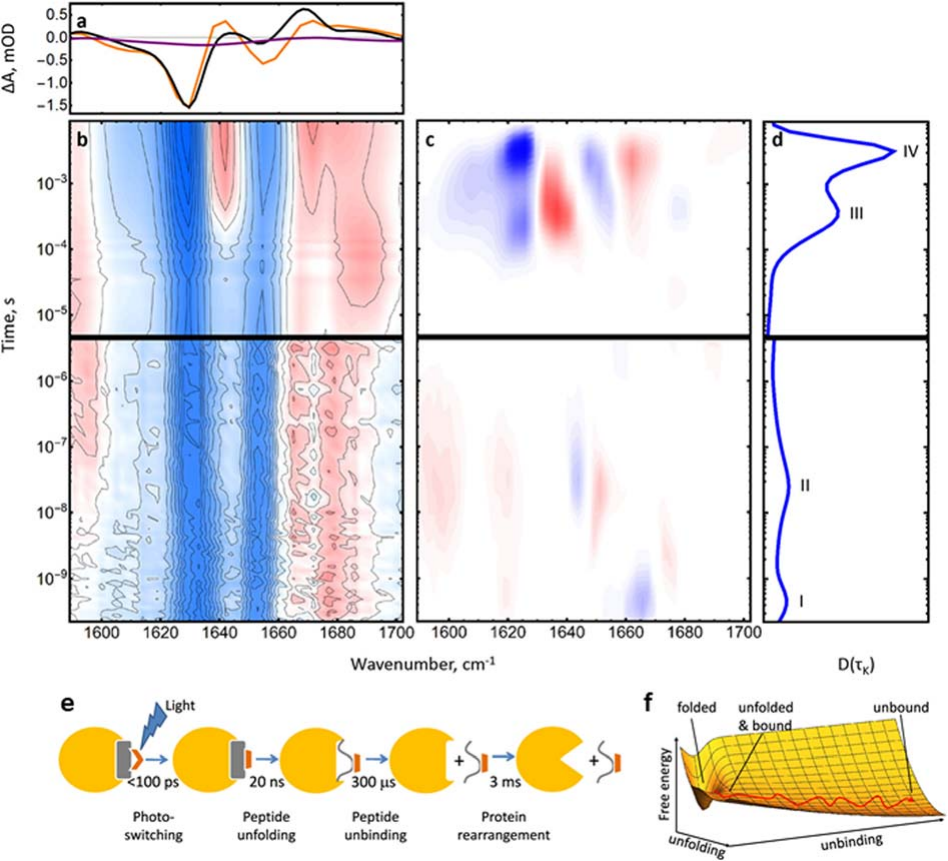
(a) FTIR difference spectrum of the complete RNase S complex (black) and the last kinetic trace at 10 ms (orange) in the amide I region. The purple line shows the FTIR difference spectrum of the S-peptide alone for comparison. (b) Transient IR spectrum of the amide I band. (c) Lifetime spectrum of the data in (b) and (d) its averaged dynamical content. (e) RNase S coupled unbinding and unfolding upon photoswitching the S-peptide conformation reveals four major processes and spans more than 7 orders of magnitude in time. (f) Sketch of a free energy surface exemplified for two orthogonal reaction coordinates for S-peptide unfolding and S-peptide unbinding, together with a possible pathway. Adapted with permission from Jankovic, B. *et al.*, *J. Phys. Chem. Lett.*, 2021, **12**(21), 5201–5207. Copyright 2021 American Chemical Society.^71^

One key feature of the reported studies of PDZ and MCL-1 systems was the fact that the perturbation induced by the photoswitch did not ultimately induce a change in the bound nature of the complexes, albeit that a significant change in structure was achieved.^65–70^ This was overcome in a study using the RNaseS complex.^71^ The latter is formed from the binding of the so-called S-protein with a smaller S-peptide which has become a well-characterised model system for protein–peptide interactions. Once again, the azobenzene photoswitch was employed to alter the structure of the S-peptide while in the bound state, leading to unbinding of the peptide from the larger protein. The complete process was studied using time-resolved IR spectroscopy covering timescales from 100 ps to 10 ms, revealing three clear timescales. Firstly, the peptide was observed to unfold on 20 ns timescales. Despite unfolding, the peptide still maintained contact with the binding pocket and unbinding was observed only after 300 μs. A further delayed process, occurring 3 ms after the initial excitation of the photoswitch, was assigned to rearrangement of the protein following the unbinding event (Fig. 4). In terms of mechanistic insight, this study found that the protein–peptide interaction involved elements of both induced fit and conformational selection limits, showing that the ability to observe processes with structural detail in real time is invaluable in being able to understand the detailed steps occurring and thus highlighting situations where current models represent limiting conditions that may be combined to a degree in real systems.^71^

A different light-activated approach to studying intermolecular interactions exploited a photoacid to deliver a pH jump^72^ to initiate folding of a leucine zipper peptide.^73^ This approach used caged proton release and demonstrated methods to maximise the local pH jump, which can be limiting in such experiments. Folding dynamics on microsecond timescales were observed *via* a combination of twin synchronised Ti:sapphire lasers to reach 40 μs and a second instrument combining a Nd:YAG pump laser with Quantum Cascade Laser (QCL) probing to reach 750 μs. While QCLs offer many advantages in terms of brightness and long timescale accessibility, their use is somewhat limited by their narrow bandwidth output which limits measurements to single frequencies.

#### Photoactive proteins

Ultrafast spectroscopies employing UV or visible wavelength excitation are ideally suited to the study of naturally occurring light activated proteins.^53,55,74–82^ This has been a rich field of study and the intention is not to provide a comprehensive review, but to focus on the impacts of extended temporal range experiments and advanced probing methods.

An example is given by a recent study using 2D-IR spectroscopy, following light activation of a cyanobacteriochrome protein with histidine kinase functionality.^83^ In this study a high pulse repetition rate (100 kHz) laser system was used to generate the IR probe pulses alongside a 15–30 Hz repetition rate Nd:YAG laser providing the visible excitation pulses. This configuration allowed collection of 2D-IR spectra on timescales from μs to ms after excitation, complementing an earlier study of the same system using synchronised Ti:Sapphire lasers to study the dynamics from nanosecond to microseconds *via* 1D-IR probing.^84^ The application of 2D-IR spectroscopy on longer timescales allowed new insight into the spectroscopic coupling patterns between vibrational modes which changed during the photocycle of the chromophore. In particular, couplings between chromophore and protein were identified and assigned to intermediate states along the reaction profile in combination with computational simulations.^83^

A similar strategy, employing two laser systems to cover time ranges from picosecond to hundreds of milliseconds was used to probe another cyanobacteriochrome (TePixJ).^85^ In this case time resolved IR spectroscopy was complemented with stationary 2D-IR measurements to add information to the assignments of time-resolved data. It was concluded that the chromophore undergoes two distinct proton transfer steps and several rearrangements on timescales ranging from picoseconds through to 200 ms for the final bond breaking step. The timescales for the reverse processes were also determined.^85^ The latter study highlights a strength and potential drawback for the use of 2D-IR spectroscopy in observing photoactivated processes. The information content in 2D-IR spectroscopy is extremely high with off-diagonal peaks providing insight into changes affecting the chromophore and the impact of these changes upon the protein. However, the spectra are complex with many overlapping contributions, which require a combination of detailed analysis and computational modelling to extract all of the available information. In addition, the extra IR pulses required to collect 2D-IR data, in contrast to the single IR probe pulse used for transient IR spectroscopy, means that acquisition times for transient 2D-IR data are long, bringing issues relating to sample and laser stability over longer periods. In the case of TePixJ, faster transient IR spectroscopy data was augmented by additional information gained from 2D-IR difference spectroscopy of the protein in the stable states of its photocycle.^85^ Indeed the difference 2D-IR approach has been applied in the absence of time resolved data to add information to the photocycle of bacterial phytochrome protein.^86^

Unravelling the complex combination of signals obtained in long timescale transient IR spectroscopy has led to the implementation of complementary molecular strategies. These include the use of non-natural amino acids. An example used the incorporation of fluorotyrosine residues in place of a natural tyrosine to modulate the local properties of the protein and establish the nature of the interaction between protein and chromophore in BLUF photoreceptor proteins.^87^ This demonstrates a powerful approach by which the information from dynamic IR spectroscopy can be given residue-level spatial specificity through control of specific areas of the protein and highlights the benefits of multidisciplinary studies in which advanced spectroscopy and chemical biology complement one another.

Transient IR spectroscopies covering long timescales are also showing benefit when used in combination with data from other methods. The advent of time resolved X-ray crystallography methods allows access to femtosecond dynamics and offers a different perspective on the behaviour of complex light activated protein systems. It also introduces a new set of even faster dynamics, which may control or impact upon subsequent processes. A recent study using fs–ms IR spectroscopy of a reversibly switchable fluorescent protein was able to provide insights that enabled data from X-ray, IR and UV-vis studies to be tied together into a photoswitching mechanism, *via* the development of novel data filtering processes, which accounted for technical issues relating to the measurement of small signals on varying baselines.^88^ The latter present problems in extracting dynamic information from low signal to noise ratio data, which is a regular challenge for IR spectroscopies on long timescales.

In photoactive systems the decay of signals over long times is exacerbated by the generally low intensity of signal from modes with small extinction coefficients in relatively low concentrations. Practical matters can also contribute, such as when the need to refresh samples between laser shots can lead to the excited sample flowing out of the area addressed by the probe laser on longer timescales. Indeed, this issue has motivated the design of alternative sample handling methodologies based around a stopped-flow design.^89^ The intention of the sample cell is to match sample movement in the cell to laser pulse arrival not only to refresh the sample between shots but also to limit any wasted time during the measurement and so increase the data acquisition rate.

An extension of the use of non-natural amino acids to modify protein behaviour is the implantation of local vibrational labels into proteins, which can then be used as a local spatial probe of changes following light activation. Such a method was demonstrated using SCN labels, which are added *via* cysteine mutations, incorporated into the structure of photoactive yellow protein (PYP).^90^ Changes in the frequency and lineshapes of the IR bands of the CN-stretching mode of the SCN labels provided insight into changes in solvent accessibility as the protein changed structure following light absorption. This approach was followed up with a stationary 2D-IR study of the dynamics of the labels pre and post light activation.^91^ The use of 2D-IR adds data on changes in the local equilibrium dynamics near the label, which can originate from solvent motion or electrostatic fluctuations arising from the protein structure. This dynamic insight complements lineshape and frequency data from IR absorption spectroscopy and enhances the ability to assign what can be rather subtle and contradictory responses of band frequency to factors such as H-bond strength and angle. Most recently, the PYP system has been revisited in unlabelled form using femtosecond to millisecond IR spectroscopy to investigate changes in protonation during the photocycle.^92^

One of the key advantages of comprehensive coverage of long timescales with IR data arises not just from being able to see the immediate impact of light absorption but the downstream effects which are likely to form the subsequent biological mechanism. In this respect a study of porphyrin compounds in cells provides an exciting new direction.^93^ The porphyrin complex was found to bind to DNA in solution, but in cells the time resolved IR data showed signatures assigned to changes in the amide I band of proteins following excitation of the chromophore. This was interpreted as showing that in the cellular environment the molecules were interacting with proteins, providing a new insight into molecular interactions and dynamics in cells.^93^ Perhaps the most exciting advance contained in this work is the demonstrated ability to carry out time resolved IR spectroscopy in cells, which should open the door to studies of increasingly complex biological systems.

#### Real time monitoring

An example of the application of ultrafast IR spectroscopy to study biomolecular processes on extremely long timescales while maintaining high time resolution is given by 2D-IR studies of amyloid fibril formation. The process under consideration involves the transition from short chain peptides (monomers) which form oligomers and then fibres *via* a process of aggregation. This complex mixture of diffusion, aggregation and structural evolution occurs over timescales of minutes to hours but understanding the underlying mechanism has relevance to a range of human diseases from neurodegenerative disorders to diabetes.^94^

2D-IR spectroscopy lends itself well to these studies for a variety of reasons. The fibrils and associated intermediates consist largely of β-sheet structures, though the presence of α-helical components has also been observed. The amide I band in a 2D-IR spectrum is exquisitely sensitive to secondary structure, through the frequency, shape, off-diagonal structure and intensity of the bands. These add considerable detail to the information available from monitoring the amide I band through IR absorption.^15,95–100^ Another advantage of 2D-IR spectroscopy arises from the fact that it suppresses broad background signals and so does not require solvent subtraction in the same way as IR absorption spectroscopy.^4^

To understand the evolution of peptide and fibril structure during the aggregation process, it is necessary to acquire 2D-IR spectra in real time as the fibrils form. This was made possible by the use of rapid scanning methodologies based on the use of acousto-optic pulse shaping, rather than interferometers, to generate the mid IR pulses needed for 2D-IR spectroscopy (Fig. 1b) and to control the time delays between them.^101^*Via* pulse shaping, 2D-IR spectra, with the required signal averaging, can be measured in less than a minute,^102^ though recent advances in high repetition rate lasers make sub-second spectral acquisition a practical reality for some samples. Pulse shaping technology has also dramatically simplified data analysis and enabled the use of phase cycling schemes to reduce the negative impact of scattered light from heterogeneous samples such as those featuring aggregates.^101,102^

Since these developments in 2D-IR methodology, rapid scan or ‘on the fly’ 2D-IR has been used for a number of studies of fibril formation and the access to real time data has been enhanced by isotope labelling strategies using amino acid residues containing ^13^C=^18^O. Isotopic substitution strategies are effective for two reasons.^99^ Firstly, the heavier isotopes shift the vibrational frequencies of the labelled bonds downwards, away from the bulk of the amide I band of the rest of the peptide, allowing observation of the behaviour of specific points in the structure. Perhaps more powerful though is the impact of these labels on the vibrational coupling within and between peptide monomers as they form 3D structures. This has enabled investigation of the alignment of folded peptides within fibrils and more recently the introduction of dihedral indexing,^103^ whereupon pairs of labels are introduced into a peptide. If the two labelled positions are arranged in a helical manner, then the coupling of the two labelled amide I modes leads to a positive frequency shift, while participation in a β-sheet leads to a downward frequency shift. This allowed a detailed investigation of not only helix formation in the early stages leading to fibril formation, with a subsequent shift towards β-sheet, but also a study of the role of lipid membranes, in the form of vesicles, upon the aggregation pathway of human islet amyloid peptide.^94^

As with the majority of protein studies using IR spectroscopy, these real time studies of fibrillation employed D_2_O as a solvent to avoid the strong H–O–H bending mode of H_2_O which overlaps the amide I band. However, following the demonstration of a method that allows 2D-IR spectra of the protein amide I band to be recorded in H_2_O,^104,105^ negating the need for isotopic replacement of the solvent, another study using 2D-IR to follow fibril formation has shown that the kinetics are influenced by the nature of the solvent, being slower in D_2_O as compared to H_2_O.^106^ The solvent-sensitivity of the aggregation mechanism has also been detected following a study of fibril formation under conditions mimicking blood serum.^107^ In this case, the additional molecular components of blood serum, salts, lipids and sugars were present, but the protein component had been removed by antibody depletion and the remainder had been subject to solvent exchange into D_2_O. The fibrillation structures of the fibrils formed were found to differ from standard buffer solution measurements revealing the potential impact of the *in vivo* environment.

One of the challenges associated with measuring evolving ensembles of molecules, as will often be found in biological systems, is the similarity of the spectroscopic signatures of proteinaceous species. Put simply, the best IR indicator of structure is the amide I band, which originates from the peptide backbone and so is common to all peptides and proteins. This means that contributions from different molecules in mixtures overlap and must be unravelled or otherwise differentiated. Isotopic labelling is one such strategy, which allows the experimentalist to isolate information from a species of interest. An alternative draws inspiration from the diffusion ordered spectroscopy (DOSY) methods developed in the NMR community.^108^ In the 2D-IR version of DOSY experiments the analyte mixture is flowed into a sample cell in parallel with a stream of solvent (for example buffer solution with no proteins). The natural diffusion of the molecules in the analyte then means that molecules will pass from the analyte to the solvent at a rate determined by their diffusion coefficient, which is inversely proportional to molecular weight. This diffusion axis adds a third dimension to 2D-IR spectroscopy by using molecular motion to separate out components of a mixture, enabling their spectra to be measured and relative concentrations determined.^108^ 2D-IR DOSY has been demonstrated for small molecules and proteins but also for mixtures containing monomers and amyloid aggregates.^109^ In the latter case, ‘depletion mode’ was used to measure the reduction in signal of small molecules moving out of the analysis volume, as opposed to into the fresh solvent, in order to combat the slow diffusion times of the larger fibrils.^109^ Thus, DOSY spectroscopy provides an interesting route to mixture analysis. While drawbacks relating to the slow diffusion time may restrict its use in real time monitoring and it is not yet clear whether diffusion may lead to shifts in the balance of equilibrium phenomena which control the sizes of clusters or aggregates, it will be a powerful addition to the armoury of tools that can be applied to extend and enhance 2D-IR spectroscopic analysis.

### Equilibrium dynamics

The focus so far has been on methods that enable the observation of evolving processes, such as fibril formation or those that are in non-equilibrium states following activation either *via* light or heat. There is also considerable interest in being able to measure the structural dynamics and fluctuations of protein molecules when at equilibrium over as long a time range as possible in order to gain insights into aspects of structural flexibility, local conformational change or solvent access and the role of H-bonding in defining and influencing local protein behaviour.^110,111^

#### Vibrational labels

2D-IR spectroscopy offers a powerful method for studying equilibrium dynamics *via* the use of local vibrational labels (Fig. 1b and 2). These were discussed briefly in relation to the use of SCN labels implanted into the photoactive yellow protein above, but they are widely used for protein studies. The advantage of 2D-IR lies in the ability to reveal the mechanisms and dynamics that contribute to line broadening and frequency fluctuations of a local vibrational label implanted into a protein structure. The motion of the protein or solvent environment leads to a time-dependent evolution of the frequency of vibration of the label group that is manifest as a change in the 2D-lineshape as a function of waiting time (*T*_w_, or pump-probe delay time, Fig. 1) in a 2D-IR experiment (a process referred to as spectral diffusion). This has been discussed in detail elsewhere, but briefly, quantification of the lineshape change, which can often be well-represented by exponential functions, reveals the characteristic time dependence of the local dynamics.^4,22,112,113^ For example, H-bond fluctuations and exchange lead to a characteristic ∼1 ps timescale in the spectral diffusion dynamics.

To be an effective vibrational label, the groups used must have a strong IR absorption and deliver minimal perturbation to the protein structure after implantation or be intrinsic to the protein. Examples of intrinsic labels have been used to study systems such as the hydrogenases^114–116^ or the use of small molecule ligands which bind to specific sites.^117–119^ Artificial label implantation is often achieved *via* non-natural amino acids such as azidohomoalanine,^120^ where the N_3_ unit provides a strong absorption near 2000 cm^−1^, or cyanophenylalanine which uses a CN stretching mode as the local probe.^121^ The SCN example given above exploited cysteine modification.^91^ In each case the labels absorb IR light in what is known as the transparent window of the IR, 1800–2800 cm^−1^,^111^ which is well separated from protein backbone and strong water absorptions, making them easy to observe and analyse.

A major problem lies in the relatively short vibrational lifetime of the modes of most probe groups, which are typically in the 1–10 ps range. As mentioned previously, the 2D-IR signal is dependent on vibrationally-excited population meaning that measurements are possible for timescales only around 5 times the vibrational lifetime of the label, which severely restricts our ability to observe equilibrium protein dynamics. As a result, there has been considerable activity aimed at making new labels with longer vibrational lifetimes in order to extend the practical reach of these equilibrium studies. This has met with some success, notably *via* the implantation of heavy atoms into the structure of probe units, which serve to block or restrict intramolecular vibrational relaxation processes and so increase the vibrational lifetime. Examples include the aforementioned SCN, which extends the lifetimes of CN groups towards 30 ps in H_2_O solutions^91^ and which have recently also been extended for use in carbohydrate derivatives.^122^ It has been shown that the vibrational lifetime can be extended further *via* the selenocyanate derivative of phenylalanine, producing a 76 ps lifetime in H_2_O.^123^

Other promising examples have produced eye-catching values of the vibrational lifetime, such as 50 ps, for a silylacetylene, which combines strong absorption with the blocking action of the Si to produce a novel probe with a long lifetime.^124^ Phenylalanine derivatives using a CH_2_SeCN group have delivered reported lifetimes greater than 200 ps,^125^ while aromatic selenocyanate derivatives have produced lifetimes on the order of 500–1200 ps,^126^ which raises the exciting prospect of observing ns-timescale equilibrium dynamics. While such progress shows great potential, the important step of implanting these new probes into protein structures is yet to be taken in most cases while many of the longer lifetimes are derived in organic solvents. The IR absorption spectrum of H_2_O features a broad combination band located at 2100 cm^−1^, which, while not intense, does offer a route to significant acceleration of vibrational relaxation if it is resonant with the vibrational mode of the probe group. This has been demonstrated for thiocyanate ions^127^ and metallocarbonyls^128^ and will be expected to impact many of these probes if they are solvent exposed when in a protein environment. While this may ultimately limit the accessible dynamic timescales achievable in some applications, such probes would be expected to provide a very sensitive measure of local hydration and solvation and be extremely effective in hydrophobic locations.

#### Electronically manipulated vibrational labels

An alternative strategy to chemical manipulation of potential vibrational probe groups may lie in the Vibrationally Promoted Electronic Resonance (VIPER) 2D-IR method.^129^ This approach employs an additional UV or visible pulse that is tuned to be off-resonance and slightly to the red of an electronic transition of the vibrational probe group or molecule (Fig. 1f). In contrast to methods discussed earlier, this UV-vis pulse arrives at the sample after the two IR pulses which constitute the ‘pump’ part of the 2D-IR pulse sequence. The result is that the IR pulses create an excited vibrational population of the probe group in the normal manner, but the excitation now means that the UV-vis pulse can excite these molecules into an excited electronic state because the IR excitation compensates for the slight initial de-tuning of the UV-vis pulse from the electronic transition. Formally, the vibrational excitation shifts the electronic transition into resonance with the UV-vis pulse by coupling the electronic transition to the excited vibrational mode. In terms of the 2D-IR experiment, the third IR pulse, which completes the 2D-IR pulse sequence, follows the UV-vis pulse to measure dynamics in the normal manner (*cf.*Fig. 1b and f). The practical difference between VIPER 2D-IR and normal 2D-IR is that by promoting the excited vibrational probe groups to an electronic state, the 2D-IR signal decay is determined by the relaxation of the electronic state, rather than the vibrational state as is normally the case.^129^ Thus, while the ground state vibrational lifetime may have been in the ps regime, electronic states can exist for microseconds or longer, massively extending the timescale over which equilibrium dynamics can be observed. An example of the potential of the method was demonstrated using the 2-isopropylthioxanthone molecule, which undergoes intersystem crossing to a triplet state upon electronic excitation, leading to a lifetime of 880 ns, while a quantum yield for intersystem crossing of 0.86 ensures strong signals.^129^ This probe was demonstrated by measuring ionic liquid dynamics over 700 ps, a value limited by optical delay stages used in the experiment and which could be significantly extended by the type of synchronised laser apparatus discussed in preceding sections.

In terms of extension to protein systems, as with all experiments there are advantages and disadvantages of the VIPER approach. The necessity of probe groups to use electronic transitions will mean that they are bulkier than the type of vibrational labels used hitherto, meaning that implantation may be more challenging while perturbation of the system may be greater. The numerous studies using azobenzene photoswitches referred to above have shown that this can be achieved with minimal disruption of protein behaviour, though the nature of the use of vibrational probes to study equilibrium structural dynamics mean that they are often desired to be in key regions of the structure where perturbation may be undesirable. There will also need to be careful accounting for the effects of any heat deposition into protein structures following the electronic excitation, while the use of an extra laser pulse means that the experiment is formally a 5th order nonlinear spectroscopy experiment, bringing with it additional potential complications for the data analysis. However, all of these issues will be more than offset by the impressive long timescale information that will become accessible *via* this method.

An evolution of the VIPER method, which uses the VIPER pulse to selectively excite different leaving groups within a molecule has also been demonstrated.^130^ This brings further potential for probing biological systems by using VIPER to activate photocaged species which may then react or otherwise initiate intermolecular interactions.

### Increasing sensitivity

In each of the experiments described above, one of the major limiting factors is the relatively small size of the measured signals. This arises from a range of factors including relatively low practical concentrations of proteins to prevent aggregation, the weak nature of IR absorptions in comparison to electronic transitions and the relatively poor performance of IR detectors when measured against those available in other regions of the electromagnetic spectrum. As a result, any improvement in sensitivity or noise reduction method can bring benefits and increases in the effective timescale over which experiments can be performed.

Significant advances have already been described above including the advent of high pulse repetition rate Yb-based lasers and pulse shaping.^131^ While associated developments in hardware continue to bring improvements in available technology.^132^ This is complemented by improvements in data acquisition methods and data pre-^133,134^ and post-processing^135^ to limit the effects of baseline fluctuations as well as techniques to improve background subtraction^136^ and to normalise data^104^ to enable accurate comparisons between different samples.

Significant improvements in sensitivity could be realised if signal enhancement approaches utilised in other areas of spectroscopy could be transferred to the IR. Efforts have been made to exploit plasmonic surfaces to enhance signals with some success.^137,138^ However, plasmonic surfaces are limited by the short-range effect of the enhancement, which means that analyte molecules effectively have to be tethered to the surface or otherwise deposited upon it in order to benefit from the enhancement. This restricts the usefulness of such methods for biological investigations as the effect of the surface and crowding need to be accounted for when analysing data.

Recently, surfaces based on Si nanostructures have shown potential to enhance signals from dilute solutions by an order of magnitude relative to IR absorption.^139^ Such structures have a greater effective penetration depth of the enhancement into the sample (micron *vs.* nanometre) bringing potential applications for protein solutions within reach. Such systems exhibit a narrower resonance than observed for plasmonic structures however, restricting the size of the portion of the spectrum that can be enhanced. Practically, this should not limit studies of vibrational probe groups which focus on single bands of relatively narrow linewidth. In addition, enhancement factors would be expected to increase significantly for nonlinear spectroscopy applications in comparison to linear absorption spectroscopy.

## Conclusions and outlook

Recent years have brought considerable improvements in our ability to observe biological processes over many orders of magnitude in time, but with high time resolution and highly localised spatial resolution. The combination of new laser technology, with manipulation of laser pulse sequences and molecular tools now offer the IR spectroscopist a broad palette of approaches to interrogate any biomolecular system.

The story will continue to evolve however as each advance brings a new range of challenges. The ability to observe long timescale biological processes now brings into reach multistep events, but ultimately these processes may be non-reversible, which brings challenges relating to making measurements with small volumes of precious sample while refreshing the sample volume between laser shots. Solutions are beginning to be found such as novel unidirectional sample handling methods for example.^140^

The ability to observe processes over long timescales brings additional challenges relating to data handling and processing. A range of methods have been used to fit and quantify data spanning long timescales, but it remains to be seen whether applications of artificial intelligence or machine learning can be used to extract more detail from these datasets than has been possible so far. Such practices will only benefit from the large quantities of data that it is possible to acquire with high pulse repetition rate laser systems.

While ultrafast laser technology is still expensive and, in general, available to only a small number of groups worldwide, the increases in speed and sensitivity, as well as the robust, turnkey nature of new equipment, should begin to make the data more accessible to researchers from other disciplines. In this respect, demonstrating complementarity of IR tools with established methods will be important in disseminating the abilities that time resolved IR can now confer. Recent examples including combining 2D-IR and NMR for multiscale studies of dynamics of relevance to cytokine mechanisms^141^ and linking time resolved IR with fs-X-ray crystallography data^88^ have shown the potential reach and value of such approaches while it is encouraging to see advanced IR spectroscopy studies increasingly contributing to broader-based studies and supplementing research chiefly based in other disciplines such as chemical biology.^94,96,142^ The field should embrace such opportunities as they bring new challenges which ultimately drive technique development, but also broaden the user base for ultrafast IR capability which will accelerate integration of the methods within the biomolecular research community. A viable route through which this may proceed is offered by proof of concept studies in cells^93,143,144^ and the development of multidimensional IR imaging modalities.^145,146^

One area that has the potential to significantly enhance the reach and impact of long timescale IR spectroscopies is the interaction with computational simulations. Ultrafast spectroscopy and molecular dynamics simulations make excellent partners, due to the similarity of the time resolution of the former and the typical time-steps made by the latter.^147^ It is however challenging to expand such simulations continuously out to the long times reached by new spectroscopy experiments, because of the computational cost and this problem is exacerbated in studies of large proteins or intermolecular interactions. It is hoped however that the quality of experimental data accessible will continue to inspire advances in related fields such as computational simulations.

Finally, it is clear that advances in laser technology over the last 25 years, since the first 2D-IR experiment,^148^ have been central to expansions in capability. It is anticipated that this will continue, making ever more ambitious experiments possible and adding further scope to the ability of IR spectroscopy to bring bond-level insight into biological processes occurring over real timescales.

## Author contributions

The work, in its entirety, was conceived and carried out by NTH. The manuscript was written by NTH.

## Conflicts of interest

There are no conflicts to declare.

## Supplementary Material
